# Electrical Execution

**Published:** 1890-10

**Authors:** 


### ELECTRICAL EXECUTION.

After every legal delay which attorneys could devise, New York's experiment of execution by electricity took place on August 6, with William Kemmler as the victim. There have been numerous secular and professional objections to the method, because the execution did not go through without some hitch in the details. The Editor of the *Record*, considering the matter editorially, does not see that this method of execution is in any way an improvement over hanging, and says the arrangements are much more horrible and trying to the victim. He concludes this editorial with the prediction that public opinion (in New York) will soon banish the death-chair as it has the rope, and imprisonment for life be the only proper penalty administered to the murderer. Whether the fact that horrible executions seem fit only for barbarians, that the poor, the ignorant and the friendless mostly serve as objects for legal vengeance, while the influential and wealthy are able to drag out the long routine of courts and finally, save a few who are popularly convicted, escape the penalty; and whether the fact that an executed man is beyond pardon, in case later evidence of innocence appear, whether all these tend to the abolition of capital punishment, time alone can demonstrate. In the abstract, with ideal courts and criminals, capital punishment seems to us but just and appropriate.

Execution by any other method, when first performed, must have been more or less bungling. Even to-day we may hear of a hangman's noose failing to close, and a struggling, horror-stricken creature, being drawn back to the scaffold, the rope re-adjusted and the trap sprung the second time. Why does not some bold humanitarian suggest execution by means of a small hypodermic injection of hydrocyanic acid, or if we seek gentle and painless death, without the horrible preliminaries, why not chloroform the victim, and then gently send him into eternity?

We give below Dr. Shrady's report of the post mortem examination, and an article by Dr. E. C. Spitzka, on page 460, one of the physicians present, the first account published in the New York *Medical Record* of August 9, and Dr. Spitzka's article being translated for THE JOURNAL, from the New York *Medicinische Monatsschrift*, of August, our excuse for this being that our readers may feel some interest in the effects of electricity as seen in this case.

---

### REPORT OF AUTOPSY ON KEMMLER.

The autopsy was held three hours after death, under the supervision of Drs. Carlos F. MacDonald, E. C. Spitzka, and George F. Shrady, of New York, and performed by Dr. W. T. Jenkins, of New York, assisted by Dr. Clayton M. Daniels, of Buffalo, N. Y.

Dr. Shrady gave out the following as the results of the autopsy so far, as information for public use: Body fairly well nourished. Rigor mortis marked, particularly in the muscles of the jaw, neck and thorax, and gradually extending from above downward, involving the feet and legs last. The post mortem hypostasis marked over lower portion of body and extending up as far as the anterior axillary line, also on the pendant surfaces of the upper and lower extremities. The upper extremities are partly fixed and rotated outward, the nails showing post mortem lividity. There was a seminal discharge, which on microscopic examination was found to contain a large number of dead spermatozoa. There was marked discoloration of the forehead about an inch in width, corresponding with the position of the strap, beginning at the hair line on the left side and extending to the hair line on the right side. A corresponding discoloration from the pressure of the chin-strap was also noted. There was an oval depression of the scalp upon the vertex, beginning at the anterior hair line and measuring four inches in its long and three and a half inches in its short diameter. Anterior to the posterior portion of the depression, and in the immediate line, there was a burn one and one-half inch in length and half an inch in width, superficial in character, slightly scorching the hair and crescentic in shape. On small of the back, corresponding to the level of the fourth sacral vertebra, below and second above, four and one half inches in vertical diameter and four and one-half inches in transverse diameter, was a burn presenting four concentric zones, of which the outermost had a pale area, corresponding to that of the rubber cup of the electrode and one-fourth of an inch in diameter.

Succeeding this was a partial and complete vesication, partial below and complete above, about an inch in diameter above and one-third of an inch below.

Then follows a transition zone, which is in its upper third a complete eschar, black in appearance, and in its lower part shows desiccation and is of a greenish-brown color. An internal zone shows a number of vesicles, chiefly peripheral, and below the centre a black eschar, half an inch in its vertical and five-eighths of an inch in its transverse diameter. Above is a tongue-shaped pale area with a lateral projection to the left of the median line, extending about two inches, and an upper projection in the dorsal furrow, which is more sharply pointed and which on its periphery shows a reddened portion with here and there vesication. In addition the back showed a number of depressions, produced by the folds of the shirt and suspenders, such as are commonly found in dead bodies lying on the back.

On incising the skin over the sternum the blood which escaped was unusually dark and fluid, and remained so on exposure. There was no vermicular action of the intestine on exposure to the air or on irritation. The diaphragm extended from the fifth intercostal on the left and the fourth on the right. The blood from the cut surface of the liver was of a crimson-like color. Abdominal organs were normal in position and appearance. The muscles of the thorax were of the usual color. Tar-like spots were noticed on the posterior border of the lower lobe of the left lung. Over half of the lung floated when placed in water, showing a marked emphysematous condition. The bronchi were normal in appearance and contained mucus and air-bubbles. The right lung was adherent throughout to the diaphragm. In the middle lobe of this lung there were numerous well marked tar-like spots. The spleen was normal in size and appearance. The left kidney weighed three and one-half ounces and the right kidney three ounces; both were intensely congested. The stomach contained a pint of undigested food. The gall-bladder was distended with bile. The heart weighed five and three-fourths ounces, valves were healthy. Bladder contracted.

The scalp on being removed showed the vertex of the skull to be in a desiccated condition, corresponding with the contact of the electrode as previously noted, but of larger area, being four inches by four inches, the zone of the scalp being only two and a half by three inches, the long diameter being antero-posterior. On removal of the skull cap the dura was normal in texture, somewhat dull in color, particularly over the area corresponding with the zone of contact. In the prerolandic region the meningeal vessels, measuring along the convexity antero-posteriorly four inches on the left side and three on the right, were filled with carbonized blood. On the internal aspect of the calvarium the meningeal vessels in the dura and their contents appeared to be black and carbonized. The carbonized vessels were so brittle that their ends were torn off with the calvarium and presented a broken, crummy appearance. The carbonization was limited in an abrupt manner. The other meningeal vessels contained blood of a crimson-like hue, corresponding to the outer burn previously described. In its narrowest portion, was seen, a little posteriorly in the median line, a dark discoloration sending out a right lateral prolongation three-fourths of an inch in the direction of the longitudinal sinus, and in width seven-eighths of an inch. Over the left hemisphere, one-third of an inch left of the median line, there was a deep carbonized spot corresponding with the carbonized portion of the calvarium. This charred spot corresponded to the dull-colored areas previously described. The pia and gyri themselves were of a pale buff color; the rest had the ordinary rosy injection of the ordinary cortex. While observing this anæmic area it was noticed that its blood-vessels began to fill. The pia and arachnoid on the convexity were perfectly normal. An interesting fact was observed on handling the pons and medulla, in that they were found to be warm. By a thermometer inserted in the fourth ventricle, the temperature was noted 97° F. This corresponds with an area temperature on the back of the neck, which was noted as 99° F. two hours after death, and 97½° F. three hours post mortem. The smaller vessels of the pia were ectatic. Capillary hemorrhages were noted in the floor of the fourth ventricle, and the same condition in the third ventricle and the anterior portion of the lateral ventricle. The perivascular spaces appeared to be distended with serum and blood. The brain cortex in the area of contact was sensibly hardened to one-sixth of its depth, where there was a broken line of vascularity. The vessels of the corpus striatum showed enlargements in different parts of their ramifications. The pons are slightly softened. The burned integument of the back, on being removed, showed the spinal muscles underneath to be cooked, like "overdone beef," throughout their entire thickness. The spinal cord was removed entire, but showed no gross appearance of pathological condition. Portions of its structure as well as those of brain-tissue, were preserved by members of the staff for purposes of hardening and microscopical examination. The blood taken immediately after death showed under the microscope a markedly granular condition, almost suggesting an electrolytic dissolution of the red corpuscles.—*From New York Medical Record.*

---

### THE TRI-STATE MEDICAL ASSOCIATION OF ALABAMA, GEORGIA AND TENNESSEE.

The next meeting will be held in Chattanooga, October 14, 15 and 16. Headquarters at the Southern Hotel.

Reduced rates on all railroads on the certificate plan (except the C. R. & C., on which round trip tickets may be purchased). Rate one fare coming and one-third returning. From some points it will be advantageous to ask for tickets to Lookout Mountain. From other points harvest excursion tickets will be on sale on the 14th. The following is the programme:

TUESDAY, OCTOBER 14TH.

9 to 10 A. M—Registration and hand-shaking.

10 to 12 A. M—Reports of Executive Committee and of the officers; reading of papers.

Afternoon Session—Reading of papers.

Evening Session—Reading of papers.

WEDNESDAY, 15TH.

Morning Session—Reading of papers.

Afternoon Session—Election of officers.

Evening Session—Address of Welcome by Geo. Robt. L. Taylor.

Response—President's address, Dr. J. B. Coran, of Tullahoma, Tenn., "The Doctor."

THURSDAY, 16TH.

Reading of papers.

The following is a list of papers so far as received:

President's Address..........................The Doctor.
J. B. Coran, M. D., Tullahoma, Tenn.

Amputation of the Hip in "New Times Method."
Duncan Eve, M. D., Nashville, Tenn.

Report of Case of Ulceration after Exsection of the Breast.
L. G. Dozier, M. D., New England City, Ga.

Report of a Case of Fracture of the Pelvis—Presentation of patient. W. T. Blackford, M. D., Graysville, Ga.

Case of Remarkable Injury with Recovery—Presentation of patient. E. A. Cobleigh, M. D., Chattanooga.

Report of Case of Fracture of the Elbow Joint.
Andrew Boyd, M. D., Scottsboro, Ala.

Abscess of the Liver.
Richard Douglas, M. D., Nashville, Tenn.

Case of Abscess of the Liver.
J. R. Rothwell, M. D., Chattanooga.

Cases of Gall Stones.
E. E. Kerr, M. D., Chattanooga.

The Dynamics of Mediumism.
J. E. Purdon, Cullman, Ala.

---

Chas. W. Tangeman, Cincinnati, O.

A Contribution to the Study of the Continued Fevers of the South. Llewellyn P. Barber, M. D., Tracy City, Tenn.

A few Remarks on the Fevers of Middle Tennessee and their Treatment. J. C. Shapard, M. D., Winchester, Tenn.

Some Phases of Typhoid Fever as well as the Abandonment of the Typho-Malaria.
J. W. Russey, M. D., Rising Fawn, Ga.

Dilated Cardiac Hypertrophy with Nephritic Complications.
W. C. Towes, M. D., Chattanooga, Tenn.

Uterine Fibroma.
J. B. Murfree, M. D., Murfreesboro, Tenn.

Some Irregular Forms of Epilepsy with Report of Cases.
W. C. Maples, M. D., Bellefonte, Ala.

Diagnosis of Corneal Affections—Fluorescence.
Frank Trester Smith, M. D., Chattanooga.

Eye Strain.
A. G. Sinclair, M. D., Memphis, Tenn.

Report of a Case of Gangrene of the Leg.
W. L. Stephens, M. D., Dayton, Tenn.

Neuralgia.
W. L. Gahagan, M. D., Chattanooga.

On all Sides a Learned Doctor.
James E. Reeves, M. D., Chattanooga.

Expert Testimony.
Mr. Sidney B. Wright, Chattanooga.

Morbid Reflex Neuroses Amenable to Surgical Treatment.
Willis F. Westmoreland, M. D., Atlanta, Ga.

Physiological Functions of the Nose.
A. B. Thrasher, M. D., Cincinnati, O.

Report of a Case of Phlegmonous Abscess.
C. H. Holland, M. D., Chattanooga.

Report of a Case of Cancrum Oris.
W. P. McDonald, M. D., Hill City, Tenn.

---

F. W. McRae, Atlanta, Ga.

Palliative Treatment of Fissure of the Anus, and Stricture of the Rectum. John P. Furniss, M. D., Selma, Ala.

Some Affections of the Rectum.
L. J. Krouse, Cincinnati, O.

Urethral Stricture and Its Complications.
J. D. Gibson, M. D., Birmingham, Ala.

The exhibits will be an attractive feature of the meeting. Fifteen firms have already engaged spaces, and the indications are that all of the available space will be taken.

FRANK TRESTER SMITH, Secretary.

*Chattanooga, September 11, 1890.*

---

TREATMENT OF OBSTRUCTIVE DYSMENORRHŒA.—(Proceedings of the International Medical Congress.) Dr. T. More Madden, of Dublin, read a short paper on this subject, and exhibited an instrument which he had devised for the rapid dilatation of the cervical canal. This instrument differs from other dilators in several respects, and especially in one which the author considered most important, *viz.:* In producing expansion of the canal from within outward; in other words, in imitating the natural process of expansion from the uterine cavity downward to the os uteri; whereas, most other dilators, such as Hegar's, etc., act in the opposite direction. In his own hands the utility of this instrument, the expansion effected by which may be measured by the affixed index, had been fully tested in a very large number of cases of sterility and dysmenorrhœa in hospital and private practice. The dilator, which does not occupy more room than the ordinary sound when introduced, may also be used, Dr. Madden said, with advantage for the dilatation of the female urethra in many cases in which this procedure is indicated.—*Record, September 6, 1890.*

### ELIGIBLE VEHICLES FOR QUININE.

Doubtless every pharmacist and physician has his favorite method of disguising the taste of unpalatable drugs, but not every one is aware that the enterprise of manufacturing pharmacists now offers such a variety of vehicles from which to choose.

Some rely almost exclusively on pills and capsules, whereby the drug is smuggled into the stomach without recognition by the gustatory nerves. But there are patients whose apparatus for deglutition is so constructed as to render it almost impossible for them to swallow pills or capsules, as well as cases where it is all important to secure immediate absorption of the medicine. When given in pill form, no matter how soluble the mass, an appreciable time must elapse before the remedy begins to have its effect.

How then can we administer quinine in solution or suspension —particularly to children or delicate ladies—without causing a disturbance in the family every time a dose has to be given? To children quinine may often be given advantageously by inunction, and the oleate of quinine especially, applied to the surface in this way, is readily absorbed and produces promptly the characteristic effect of the drug. Suppositories must not be forgotten in cases where the stomach is particularly irritable, and the hypodermic injection presents itself as a dernier resort when a prompt and powerful influence is required. But in ordinary cases quinine may be administered by the mouth.

One plan is to mix the quinine with some alkali or astringent, so that the bitter sulphate or muriate becomes converted into the tasteless alkaloid or tannate.

Another plan is to combine with the quinine a mixture having a bitterness of its own, which shall blend with and modify the intolerable bitterness of the quinine, some aromatic being generally added to still further disguise the objectionable taste.

It is on this principle that cascara cordial operates, and many of those who have tried this vehicle, declare that it is the best that has yet been offered. The especial advantage which it possesses over all others is the fact that it is a laxative agent, and so renders more efficient the action of the antiperiodic.

Licorice has been long known as having a remarkable power of covering the taste of bitter medicines. This property is due to a peculiar principle called glycyrrhizin, a glucoside, insoluble in water and in acid solutions, but readily dissolved by the aid of alkalies.

Where quinine is given in powders, it may be rendered nearly tasteless by simply rubbing it up with a small quantity [one fourth its weight] of ammoniated glycyrrhizin [ammonium glycyrrhizate].

"Fluid extract licorice, for quinine mixtures," is one of the most efficient of all the preparations employed for covering the bitter taste of quinine. The best way to use it is to drop a dose of the powder into a little of the fluid extract contained in a spoon, mix it thoroughly and swallow at once.

Aromatic elixir of licorice is to be used in the same way as the fluid extract, but is especially useful in the drug-store when a single dose of quinine is called for to be taken at once.

Yerba Santa contains a principle analogous to glycyrrhizin, which renders quinine in its presence as tasteless as starch. It appears to act like glycyrrhizin by producing a peculiar impression upon the gustatory nerves: it does not, as stated by some, produce with the quinine an insoluble compound. Unless the mouth is thoroughly rinsed after taking the mixture, a bitter taste will gradually develop as the nerves recover from the influence of the Yerba Santa.

To some parsons the taste of Yerba Santa itself is disagreeable, and when this is the case licorice is to be preferred. Barring, however, idiosyncrasy in this respect, we can recommend the preparations of Yerba Santa as the best means of rendering quinine tasteless. Aromatic syrup of Yerba Santa renders it possible to give little children full doses of quinine without the vigorous remonstrances which physicians and parents have learned to regard as inevitable.—*Northwestern Pharmacist.*

### NEW TREATMENT OF TUBERCULOSIS BY THE VACCINE METHOD.

On November 19th last Drs. J. Grancher and St. Martin addressed to the *Academie de Medicine* a sealed packet relating to a method of treatment and preventive inoculation of tuberculosis based upon numerous experiments which they had made on rabbits. The communication made by Dr. Koch to the Berlin Congress (of which the full text was published in the *British Medical Journal* of August 16th), concerning the results which he has obtained in rendering guinea-pigs refractory to tuberculosis, or in curing them of advanced forms of tuberculosis, has induced MM. Grancher and Martin to make known their researches on the same subject earlier than they would otherwise have done. In all these experiments they chose the rabbit as the subject of inoculation and intravenous injection, because there is thus produced a tuberculosis which kills very quickly, and at an almost fixed date, with constant lesions of the liver, the spleen, and the lungs, and which defies all local treatment. Tuberculosis thus induced being always fatal, a solid basis is thus secured which allows exact appreciation of the negative or positive results of any method which tends to produce the refractory state or to cure after infection. The method employed by MM. Grancher and St. Martin was the injection of tuberculous cultures attenuated in various degrees, and used like the dried spinal marrow in Pasteur's treatment of rabies and hydrophobia. Nine degrees of attenuation have been obtained, the four last being such that the cultivations remained sterile. The injections were made first with the most attenuated cultivations, and then with more and more virulent ones. The authors consider that by this method they have succeeded, on the one hand, in conferring on rabbits prolonged resisting power against the most certain and the most rapid experimental tuberculosis, and, on the other hand, in conferring an immunity against that disease, the duration of which remains to be determined.—*British Medical Journal, August 30, 1890.*

THUNDER AND SOUR MILK.—The effect of thunderstorms in turning milk sour is a matter of constant observation in every household. It is not certainly known to what element in the air this souring action on milk is to be directly attributed, and most people are content to ascribe it to "electricity in the air." An Italian *savant*, Prof. G. Tolomei, has lately made some experiments with the view of elucidating this question. He found that the passage of an electric current directly through the milk not only did not hasten, but actually delayed acidulation, milk so treated not becoming sour until from the sixth to the ninth day, whereas milk not so electrified became markedly acid on the third day. When, however, the surface of a quantity of milk was brought close under the two balls of a Holtz machine the milk soon became sour, and this effect he attributes to the ozone generated, for when the discharge was silent the milk soured with greater rapidity than when the discharge was explosive, in the former case more ozone being formed than in the latter. The souring of milk is generally attributed to the growth of a ferment (bacterium), which converts the milk sugar into lactic acid. It is possible, then, that the presence of ozone in the air overlying the milk hastens the growth and multiplication of the bacterium. The first observation—namely, the retardation of souring by the passage of a current through the milk—may be a point of practical importance to milk traders. Any methods of preserving milk from its first retrogressive changes, which does not involve the addition of extraneous substances (antiseptics) to the milk, and which is at the same time cheap, effective, and not likely to prove injurious to the consumer, is sure to be welcomed at a time when milk is sent long distances to market, and is often stored for a considerable time before it reaches the consumer.—*British Med. Jour., Aug. 30, 1890.*

---

THE mistake of publishing the discussion upon Dr. K. P. Moore's paper, which appeared in our last issue, was due to the fact that a galley proof of the proceedings of the State Medical Association was sent us for copy. We regret the mistake, as Dr. Moore was not aware of it until he saw his article in print.

MEDICAL LAW IN THE STATE OF WASHINGTON, U. S. A.—The Americans carry their love of protection into matters medical as well as other things, and accordingly a man who has obtained his diploma in one State cannot practice in another without passing a fresh examination ; the number and diversity of the diploma-granting bodies are no doubt a sufficient justification for protective measures. A letter from a successful candidate shows how this system works in the State of Washington, and does not add much to the reputation for common sense which the Americans are supposed or suppose themselves to be so largely possessed of. The examining board is composed of four regular practitioners, three homœopaths, a "physio-medic" and an "eclectic ;" what the peculiar tenets of the two latter practitioners are, or in what way they differ from each other, we have no idea ; but their professional attainments are abundantly evidenced by the questions they asked. The writer of the letter referred to gives several of the questions, of which we may select the following as sufficient for our purpose. The "physio-medic" asked : "What does delayed dentition prognosticate ?" The candidate could not tell, nor could the two or three members of the board to whom he afterwards submitted the question. Amongst the problems—for we call them nothing else—propounded by the "eclectic," one was, "What's the *peremptory* condition of pepsin in the stomach ?" It was ascertained that the proper answer to this abstruse question was, "hydrochloric acid." Another of his problems was, "What's the effect of too much red corpuscles in the blood ?" Altogether we do not think that the board as at present constituted is likely to do much credit to the governing body which has called it into existence.—*British Med. Jour., Aug. 30, 1890.*

---

HYPOGASTRIC PUNCTURE OF THE BLADDER.—Dr. Deneffe says that too serious a view is taken of hypogastric puncture of the bladder in cases of retention. It ought not to be considered as dangerous, and as a last resource. He quotes a case of hypertrophied prostate which prevented micturition and catheterism, in which the patient was punctured seventeen times without suffering any inconvenience. The seventeenth time the trocar was allowed to remain, and ten days afterward micturition took place spontaneously through the urethra. Nevertheless, the trocar was allowed to remain twenty-nine days, when it was removed. The fistula was closed in four days, and the patient recovered permanently.

Hypogastric puncture is considered a mild operation by Dr. Deneffe, and he states that he has performed the operation on three hundred and one patients, with a mortality of only two and a half per cent. A patient suffering from enlarged prostate or stricture suddenly cannot micturate, and this is usually due to a sudden spasm of the posterior portion of the urethra being superadded to the original lesion. Catheterism is not adapted to reduce a spasmodic contraction, and the urethra affected by spasm can only be benefited by the contact of irritating urine being removed.—*Journal de Medicine de Paris.*

